# Drug Research Meets Network Science: Where Are We?

**DOI:** 10.1021/acs.jmedchem.9b01989

**Published:** 2020-04-27

**Authors:** Maurizio Recanatini, Chiara Cabrelle

**Affiliations:** Department of Pharmacy and Biotechnology, Alma Mater Studiorum—University of Bologna, Via Belmeloro 6, I-40126 Bologna, Italy

## Abstract

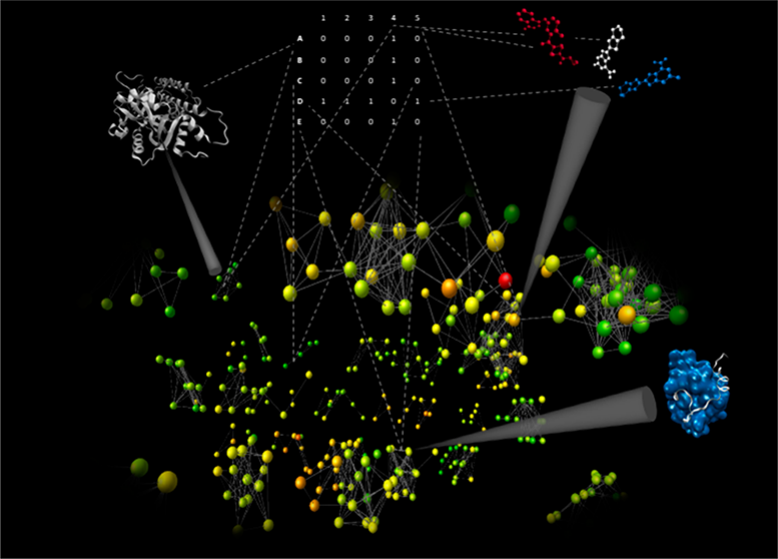

Network theory provides one of the most potent analysis tools for the study of complex systems. In this paper, we illustrate the network-based perspective in drug research and how it is coherent with the new paradigm of drug discovery. We first present data sources from which networks are built, then show some examples of how the networks can be used to investigate drug-related systems. A section is devoted to network-based inference applications, i.e., prediction methods based on interactomes, that can be used to identify putative drug–target interactions without resorting to 3D modeling. Finally, we present some aspects of Boolean networks dynamics, anticipating that it might become a very potent modeling framework to develop *in silico* screening protocols able to simulate phenotypic screening experiments. We conclude that network applications integrated with machine learning and 3D modeling methods will become an indispensable tool for computational drug discovery in the next years.

## 1 Introduction

The first decade of the 2000s has seen a consistent modification of the drug research landscape, due, among other aspects, to a rethinking of the drug discovery paradigm^1^ and to the entrance into the era of Big Data.^2^ The paradigm change in drug discovery is derived from both the need to improve the success rate of pharmaceutical industry and the diffusion across the drug research community of discoveries and concepts related to systems biology.^3^ In fact, driven by the initially debated and finally accepted proposal of multitarget drugs,^4^ the conventional one-disease–one-target idea was abandoned to embrace a less reductionist view. The inspired concept of network pharmacology elaborated by Andrew L. Hopkins^5^ allowed us to move toward a new system-based paradigm.^6,7^ On the other hand, the availability of relevant amounts of data, from molecular descriptors to bioassay results, from -omic information to clinical records, provides the starting material for the elaboration of multilevel integrated models that take full advantage of the constantly growing informatics technology for model building, analysis, interpretation, and exploitation.

Complexity is a distinctive trait of living systems, and recently researchers have started to investigate natural complex systems with adequate theoretical tools.^8^ In particular, network science permits catching of the behavior of a system as a whole, especially regarding its emergent properties, that is, the features that arise from the interaction of the systems’ parts and not as a mere sum of them. In the field of medicine, the point of view proposed by Albert L. Barabási is illuminating: if disease phenotypes can be viewed as emergent properties deriving from the interconnection of pathobiological processes, in turn arising from the cross-talk of molecular, metabolic, and regulatory networks at cellular level, a framework of “network medicine” might help in exploring causes and finding therapies at a global integrated level.^9^

Networks rely on the idea of modeling a real system as a map of interconnected dots and lines representing the set of elements and the set of relationships between them, respectively. The mathematical description of a network is addressed by the graph theory such that graph stands for network, and often, even though inaccurately, these terms are used as synonyms. Elements of the network are called nodes, connections between them, links or edges. The start of graph theory is commonly traced back to Leonhard Euler’s paper *Solutio problematis ad geometriam situs pertinentis* that appeared in 1741 on the *Commentarii Academiae Scientiarum Petropolitanae*.^10^ The problem to be solved was to find a pathway going through all seven bridges of the city of Koenigsberg without crossing the same bridge twice. Euler demonstrated the inexistence of such a pathway, based on an abstract representation of the four areas of the city (nodes) linked by the seven bridges (links). In other words, he, for the first time, used a graph to represent and solve a mathematical problem. From then on, graph theory and network science developed mainly in the fields of mathematics and physics, respectively, and today they form a sound body of science and provide formidable tools to deal with the architecture and properties of complex systems.^11−13^

Now the question is to what extent the network-based approach is influencing or has already influenced the way new therapies are looked for. Actually, the ways network theory is applied to drug discovery are numerous and aimed at different purposes. Limiting ourselves to the medicinal chemistry/drug design area, the main fields where network-based methods are employed are target identification and drug repurposing, both eventually in the context of polypharmacology. Further fields of application are the analysis of chemical spaces and the prediction of adverse drug reactions or toxicity. Several examples of these applications of network theory have appeared in the literature in the past years, and they have been reviewed in a number of papers. It is worth mentioning here the comprehensive review on the argument published in 2013 by Peter Csermely et al.^14^ that laid the foundations of a network-based drug science. In the present Perspective article, we recapitulate the basis of the approach and see how far we have gone with it by illustrating some recently published results. We also discuss some possible advancement and points of attention in the field.

## 2 Data, Databases, and Networks

### 2.1 Data and Databases

The first and most important issue to consider when dealing with the construction of networks to investigate pathobiological processes and drug effects on them concerns the material that we use to build these models, that is, what we usually call “data”. By this term we mean a wide variety of items, expressed in numerical, alphabetical, or digital form and collected in databases that often are publicly available. Although in the drug discovery community we make use of data sets at least since the early days of QSAR, the information made available by the high-throughput experimental technologies is growing at an unprecedented pace. Nowadays, we can access data sources on compounds, targets, and diseases that cover millions of molecules and thousands of proteins and genes in almost every therapeutic field.

#### 2.1.1 Chemical Databases

On the chemical compounds side, the number and variety of freely accessible databases increase continuously, and each of these data sets contains information for numbers of molecules that range from 10^3^ to 10^7^. Recently, Yang et al. attempted to classify the public chemical databases into six categories based on their content, namely, (1) chemical information, (2) bioactivity, (3) drugs, (4) natural products, (5) commercial availability, and (6) fragments.^15^ All types of data contained in the chemical databases can be useful for drug design purposes in general, but for what concerns network applications, data on bioactivity, drugs, and natural products are most interesting. With this respect, the most popular databases are CHEMBL^16^ and PubChem,^17^ which provide knowledge on bioactive compounds, particularly data from activity assays and target information. DrugBank^18^ contains data on approved and experimental drugs and can be an important source of information in target identification and drug repurposing studies. On the purely chemical side, ChemSpider^19^ is a very rich source of physicochemical and spectral data, as well as of names, synonyms, and identifiers. In Table 1, the main features of the mentioned databases are summarized.

**Table 1:** Chemical Databases

database name	description	url	ref.
ChemBL	Collection of bioactive drug-like small molecules with 2D structures, calculated chemical properties and bioactivities.	https://www.ebi.ac.uk/chembl/	(16)
PubChem	Open chemistry database of mostly small molecules that collects information on chemical structures, chemical and physical properties, and biological activities. It is structured into three linked databases: substance, compound, and bioassay.	https://pubchem.ncbi.nlm.nih.gov/	(17)
DrugBank	Freely accessible data on small molecules and biotechnological drugs (with chemical, pharmaceutical, and pharmacological profiles), and drug targets (sequence and functions of target/enzyme/transporter/carrier) intended as drugs encyclopedia.	https://www.drugbank.ca/	(18)
ChemSpider	Free chemical structures database collecting structures and related information, such as physicochemical properties and interactive spectra, made accessible through a fast search engine allowing search by name, structure, or advanced options.	http://www.chemspider.com/	(19)

The most important issue to consider when using a chemical data set is the need of preprocessing its content, a crucial operation that may take a long time but indispensable to lower the probability of obtaining misleading results and/or building erroneous models. Tropsha and co-workers have addressed this argument in the context of QSAR modeling,^20^ but their conclusions and suggestions fit perfectly here. In particular, the curation of a data set should consider chemical, biological, and item identification aspects, as, for example, representation of chemical structure and presence in the data set of nonstandardized structures (salts, ions, etc.), variability of biological data for the same compound, reproducibility of results through different laboratories, presence of activity cliffs, misspelled, or mislabeled compounds, and incorrect identifiers. The same authors provided thoughtful guidelines for chemical and biological data curation to avoid errors and to ensure the stability and reliability of the models.^21^

#### 2.1.2 Biological Databases

When dealing with target identification or drug repurposing studies in a system-wide perspective, it is mandatory to include in the network the knowledge that is increasingly generated by the huge array of biological techniques. Fortunately, the availability of free access biological databases is even bigger than that of chemical substances. The journal *Nucleic Acid Research* (NAR) in 2019 published the 26th annual issue of the Molecular Biology Database Collection,^22^ presenting an impressive list of 1613 databases, shortly describing the new entries, and eventually updating old ones. Also in this case, databases are grouped, reflecting the categorization of the databases in the NAR online database collection, that is, (1) nucleic acid sequence and structure and transcriptional regulation; (2) protein sequence and structure; (3) metabolic and signaling pathways, enzymes, and networks; (4) genomics of viruses, bacteria, protozoa, and fungi; (5) genomics of human and model organisms plus comparative genomics; (6) human genomic variation, diseases, and drugs; (7) plants; (8) other topics. It is worth noting that the above-mentioned data sources are mostly genotype-based, leveraging the fast development of the -omic technologies. Among the conspicuous number of available databases, some can be of particular interest for the drug design field, like those containing information regarding proteins, as either general sequence (e.g., SMART,^23^ UniProt^24^) or sequence of individual protein families (e.g., GPCRdb,^25^ Kinomer^26^), protein structure or protein–protein interactions (e.g., PDB,^27^ STRING^28^), metabolic and signaling pathways (e.g., Reactome^29^), human genes and diseases (e.g., DisGeNET^30^). A special mention is deserved here of two important resources that provide drug researchers with invaluable information on targets and mechanisms of action of small molecules: the ConnectivityMap^31^ and LINCS^32^ platforms that give access to gene transcriptional profiles in response to perturbation by drugs or other chemical compounds. In Table 2, the main features of these databases are summarized.

**Table 2:** Biological Databases

database name	description	url	ref
SMART	Simple modular architecture research tool is a Web tool for identification of protein domains and exploration of domain architectures.	http://smart.embl-heidelberg.de/	(23)
UniProtKB	UniProtKB consists of two databases: SwissProt (manually annotated) and TrEMBL (automatically annotated and unreviewed). UniProtKB collects protein sequences providing rich annotations.	https://www.uniprot.org/	(24)
GPCRdb	GPCRdb stores reference data on GPCRs sequence, structures, mutations, and ligand interactions. GPCRdb also provides a suite of Web tools and interactive diagrams for illustrative purposes.	https://gpcrdb.org/	(25)
Kinomer	Protein kinases database contains annotated classification of 43 eukaryotic genomes. Kinomer is accessible through a Web interface, which enables the retrieval of sequences and the classification of arbitrary sequences.	https://www.compbio.dundee.ac.uk/kinomer	(26)
PDB	Protein Data Bank is a repository of experimentally determined 3D structures of proteins, nucleic acids, and complexes with metal ions, drugs, and small molecules.	https://www.rcsb.org/	(27)
STRING	Search tool for retrieval of interacting genes/proteins (STRING) database of known and predicted protein–protein interactions, including physical and functional associations for a large number of organisms. STRING allows users to visualize interactions network and to make analysis.	https://string-db.org/	(28)
Reactome	Reactome is a relational pathway database in which signaling and metabolic molecules are organized into biological pathways and processes.	https://reactome.org/	(29)
DisGeNET	DisGeNET is a platform that integrates and standardizes data about disease associated genes and variants (628 685 gene-disease associations, GDAs, and 210 498 variant-disease associations, VDAs).	https://www.disgenet.org/	(30)
ConnectivityMap	CMap database contains gene-expression profiles from cultured human cells treated with perturbagens (bioactive small molecules).	https://clue.io/cmap	(31)
LINCS	Library of integrated network-based cellular signatures catalogues perturbation-response signatures employing a various set of perturbations, model systems, and assay types.	http://www.lincsproject.org/	(32)

#### 2.1.3 Phenotypic Data

Moving to the field of human phenotypic data, we leave the territory of drug discovery to enter into the precision medicine arena. In this context, the pervasive digitalization of healthcare is providing quantitatively very important sources of phenotypic data, like primarily those contained in the electronic health records (EHRs) but also those generated by wearable devices or apps.^33^ Limiting to EHRs, the information embedded in these documents includes the description of the health/disease status of individuals, clinical test results, drug prescriptions, and eventual adverse effects.^34^ Of course, privacy issues limit the availability of this kind of data, and we cannot find publicly accessible databases yet. Nevertheless, several initiatives already exist to collect EHRs and related data and make them available for the biomedical research, as, for example, the “All of Us” initiative.^35^ As regards the contribution of this kind of information to the drug research, we observe that integration of phenotypic and genotypic data might be a necessary step toward a deeper understanding of the biological processes at the base of the onset and progression of diseases.^36^ Even though it is not yet clear how this integration will be translated to the discovery of *new* drugs, this approach is being applied for drug repurposing, as shown by the recent work by Khosravi et al.,^37^ who proposed a list of repurposed drug candidates for melanoma treatment after the analysis of genome- and phenome-wide association studies.

### 2.2 Building the Network

Given the wide availability of data on molecules, genes, proteins, cells, tissues, and diseases and the assumption that these entities are connected and representative of a more or less complex system, one needs to build and visualize the network (actually, the graph representing the network) in view of the subsequent analysis. The computational tools available for network visualization and analysis are countless and range in complexity depending on the dimensions of the data set and of the task to be executed. There are several popular and efficient software that can work on personal computer/workstation and allow one to perform all the basic operations on the network, from visualization to analysis of the basic parameters. Cytoscape^38^ is the most popular tool, but also Gephi,^39^ Pajek,^40^ and NetworkX^41^ are rather widespread in the biological community (see Table 3 for details on the main features of the softwares). A recent review by Pavlopoulos et al.^42^ analyzes and compares the performance of different software tools for the visualization of even large networks up to the order of magnitude of 10^6^ nodes and edges. However, when the amount of data increases, the memory requirements to load large matrices become prohibitive even for powerful workstations, and higher performance computing is needed to analyze the network. A solution can be to distribute data and processes on high numbers of cores by means of frameworks, like Hadoop^43^ or Apache Spark^44^ (see Table 3). In this way, computations are partitioned across clusters of machines that work in parallel and carry out the jobs in reasonable time and with high efficiency.

**Table 3:** Network Building and Visualization Systems

database name	description	url	ref
Cytoscape	Cytoscape is a platform for visualization, analysis, and integration of networks via basic functionalities or through apps; conceived mainly for biological research.	https://cytoscape.org/	(38)
Gephi	Gephi allows the visualization and exploration of all types of large graphs in real-time through a 3D render engine.	https://gephi.org/	(39)
Pajek	Pajek enables analysis and visualization of large networks having some thousands or millions of vertices.	http://mrvar.fdv.uni-lj.si/pajek/	(40)
NetworkX	NetworkX is a Phyton package designed for the creation and analysis of structure, dynamics, and functions of networks.	https://networkx.github.io/	(41)
Apache Hadoop	Open source framework for storing and processing large data sets across clusters of computers in a distributed environment through simple programming models.	https://hadoop.apache.org/	(43)
Apache Spark	A fast cluster computing system for large-scale data processing powering different libraries (SQL, MLlib, GraphX), and easy to use interactively from the Scala, Python, R, and SQL shells.	https://spark.apache.org/	(44)

## 3 Networks to Study Systems of Pharmaceutical Interest

As a first step to illustrate the use of networks in drug research, we can consider studies aimed at visualizing and analyzing systems of biomedical/pharmacological interest. A short explanation of basic concepts of network theory and of the terminology used in this field is provided in the Supporting Information.

### 3.1 Networks for the Analysis of Molecules Data Sets

A relevant example of the use of network analysis in the small organic molecules context is that of chemical space networks (CSN), a framework proposed and developed by G. Maggiora and J. Bajorath.^45,46^ In their first review on the argument, these authors stated that “‘molecular networks’ are thought to provide an alternative way to represent and navigate chemical space” and that “chemical space exploration [...] is often motivated by the need to better understand structure–property relationships of small organic compounds”.^45^ In the initial applications of the CSN formalism, the aim was simply to obtain a new coordinate-free representation of the chemical space, based upon pairwise compound similarities, instead of the more commonly used coordinate-based representations referring to the descriptor space. In subsequent papers, Bajorath and co-workers showed how well network representations allowed highlighting of the properties of a chemical space viewed as a complex system to whom emergent properties like biological activity could be associated.^46^ Different similarity metrics were introduced and validated,^47^ and together with the analysis of the network topology parameters, they were shown to be a powerful tool to visualize and analyze the structure–activity relationships (SARs) of moderately sized compound sets.^46,48^ In this context, the analysis of the CSN through adequate metrics and algorithms can reveal the presence of communities (clusters) of compounds sharing latent characteristics not immediately evident from the common table format.

**Figure 1:**
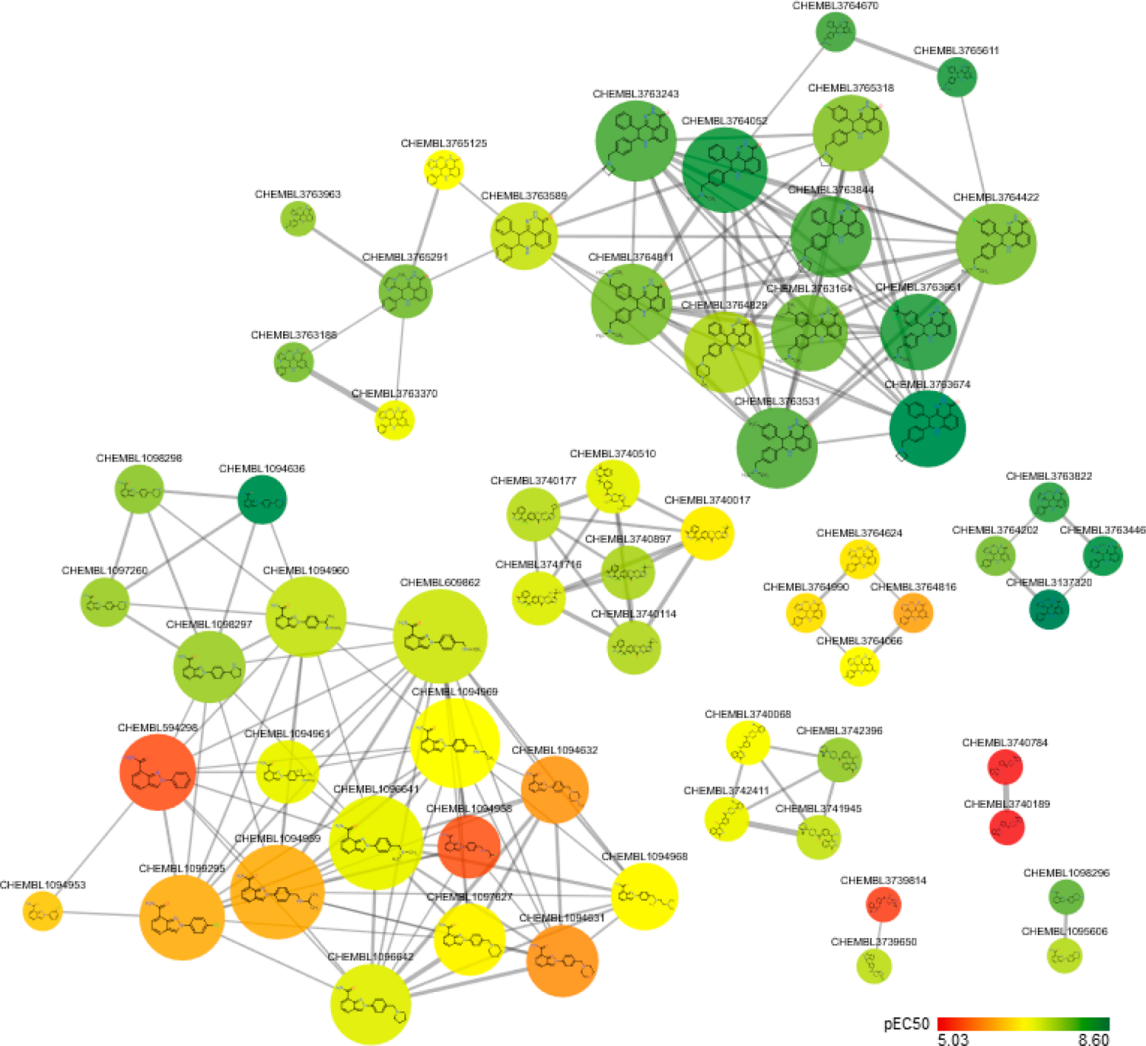
Exemplary CSN of PARP inhibitors. PARPs 1, 2, and 3 family inhibitors with a measured EC_50_ were retrieved from CHEMBL.^16^ The pairwise chemical similarities between compounds were assessed by means of Tanimoto coefficient (Tc) values calculated for the ECFP4 fingerprints^49^ of the molecules generated by Canvas^50^ (Schrödinger, LLC, New York, NY, 2019). Pairs of inhibitors were connected only if their calculated Tc value exceeded the threshold value of 0.55. The chemical structures of the inhibitors are shown inside the nodes that are colored according to pEC_50_ values ranging from red (lowest potency) to green (highest potency) and sized based on node degree from small (low degree) to large (high degree). Edges are weighted by Tc values from thin (Tc = 0.55) to thick (Tc = 1) width. The network was generated by means of Cytoscape^38^ version 3.7.2.

To illustrate a simple CSN application, in Figure 1 a network of 62 poly(ADP-ribose)polymerase (PARP) inhibitors is shown. The network accounts for relationships between the compounds, and the links among them were derived from pairwise similarity values calculated based on fingerprints. The inhibitors are represented by nodes (62) that are connected by edges (188) if their structure similarity exceeds a threshold (see the legend to Figure 1). The nodes are colored according to potency. This visualization of the chemical space based on similarity calculation facilitates the identification of the different structural families of PARP inhibitors (the main connected components of the network), and the color-coding allows one to grasp immediately the SAR of the set of compounds.

The usefulness of the CSN framework was also recently shown in the field of natural product extraction and characterization, as reported in a paper by Nothias-Esposito et al.^51^ Here, the authors analyzed the extracts from two species of *Euphorbia* plants by means of tandem mass spectrometry and generated the network representing the isolated compounds using the mass spectra as nodes linked by similarity. In particular, to give rise to the network, the spectra were converted into vectors that were used to calculate a similarity score between each pair of spectra.^52^ The network then allowed visualization of structurally related molecules (based on their spectra, irrespective of their structure) that were subsequently identified by performing a search against a public reference spectral library.

The above examples deal with relatively small data sets, but the versatility of network analysis can be exploited even when the amount of data becomes much greater. This is the case of a work recently published by Miho et al., who performed a large-scale analysis on networks built from antibody repertoires.^53^ To appreciate the dimension of the problem, it is enough to note that to build the network of a repertoire of, for example, 10^6^ clones (the nodes), one needs a similarity matrix of 10^12^ elements to define the edges. This is a relevant computational task that the authors tackled by leveraging the power of parallel distributed computing through the Apache Spark framework.^44^ The analysis of the network of complete antibody repertoires allowed overcoming of the limitations of using only portions of the network that are not *a priori* statistically representative of the properties (parameters) of the whole system and led the authors to derive some general principles on immune repertoire architecture.^53^

### 3.2 Protein Structure Networks

If we consider that a protein, like any other molecule, is an ensemble of interacting elements (the amino acid residues, in this case), it is immediately derived that it represents a complex system in which structure, dynamics, and eventually function can be viewed as emergent properties stemming from the relationships among the residues. In this context, protein structure networks (PSN) are widely studied, as the network approach is considered quite suitable to deal with the structure–function relationships, also in the light of the fast growth of analytical/biophysical technologies applicable to protein structure determination. PSNs are built by considering the amino acids (usually the Cα atoms) as nodes that are connected by a link if the distance between them falls within a cutoff value. The analysis of the parameters describing the properties of the PSNs lends itself to the study of the protein’s 3D architecture and its implications in issues like allosteric communication, folding, and model validation.^54^ In a recent review, Di Paola et al. discussed several applications of graph theory to the description of protein properties in terms of network parameters and advanced the hypothesis that the protein contact graph (representing the PSN) might in the future be considered as the structural formula of proteins.^55^ It is interesting to note that the PSN analysis can be carried out also in combination with other computational techniques to investigate the protein structural/conformational behavior. In particular, molecular dynamics simulations and eventually binding free energy calculations can be synergistically applied to studies of pharmaceutical interest. For example, Verkhivker showed the usefulness of this combined approach to interpret how allosteric effects act on the modulation of protein functions in the presence of inhibitors^56^ or cancer driver mutations.^57^ Recently, Amusengeri et al.^58^ applied a computational protocol including a residue network analysis to identify allosteric inhibitors of *Plasmodium falciparum* 70 kDa heat shock proteins as new antimalarial drug candidates.

### 3.3 Human Disease Network and Drug Discovery

Considering drug research from a system-wide, broad perspective, we cannot help but face the studies on the human disease network carried out by Barabási and his co-workers. In 2007, this author proposed the term “diseasome”^59^ to indicate the network of all genetic disorders systematically linked to all disease genes: it is an example of a bipartite network (see Supporting Information), where diseases are connected to the genes known to cause or effect them.^60^ This approach provides a wider view on diseases with respect to the usual classification, revealing possible connections between some of them, and eventually allowing for new possibilities of treatment. The same authors further elaborated on the diseasome concept by taking into consideration molecular interaction networks, i.e., the “interactomes”. The latter can be gene regulatory networks (GRNs), protein–protein interaction (PPI) networks, or metabolic networks and are indispensable further elements to consider to build a system view of the cellular mechanisms underlying the phenotype–genotype relationships in human diseases.^61^ It is evident here the effort to hold together different layers of a complex system, by leveraging the systems biology approach,^62^ in order to escape the simplifications of reductionism.

Focusing more specifically on the relevance of the above considerations in drug research, first of all, we must recall that, however, as far as drug action is concerned, the drug–target interaction remains a key event that maintains its centrality across paradigms, from the old “magic bullet” idea to the present polypharmacology, systems-based approach. Having said that, it appears clear how crucial can be the modeling of the drug–target interactomes. This goal has been pursued for a decade or more, from the early representations due to the work of Hopkins^5,63^ to the important contributions from the Barabási group.^64−66^ In essence, from the data sets of experimental information (as, for example, DrugBank, Table 1), a bipartite drug–target network (DTN) is built from which projections on the drug or the target component are obtained. This provides both the drug network (DN), and the target network (TN), each made by nodes of the same type (drugs or targets, respectively) connected to each other if they share at least one target (the DN) or one drug (the TN). Analysis of the topology of DTNs, DNs, and TNs can reveal interesting and sometimes unexpected details on how and why drugs act on certain targets or target communities within the diseasome. It can also provide useful information for the design of drug combinations, the drug repurposing, or the interpretation of toxic effects. Applications of this kind of analysis are increasingly appearing in the literature and are reviewed in recent articles (e.g., see refs (67) and (68)).

**Figure 2:**
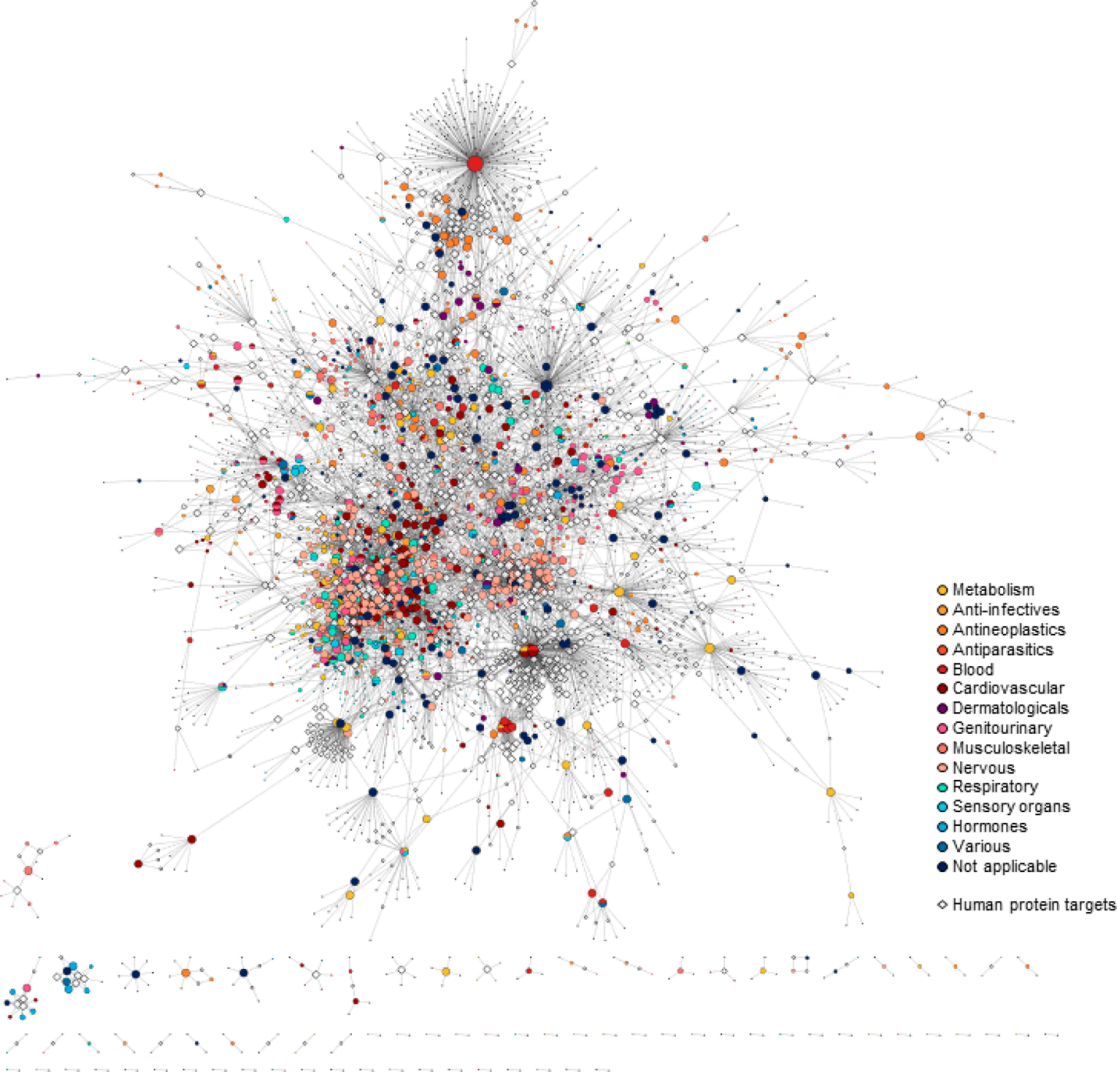
Drug–target network. The DTN was built from DrugBank^18^ version 5.1.5 retrieving the drug–target interactions between approved small molecule drugs and human protein targets. Drugs are represented as circle-shaped nodes, and protein targets are represented as diamond-shaped nodes. As shown in the inset, drugs are color-coded according to the first level anatomical therapeutic chemical (ATC) codes as reported in DrugBank. The nodes size accounts for the node degree from small (low degree) to large (high degree). Edges connect only drugs and targets nodes. The network was generated by means of Cytoscape^38^ version 3.7.2.

As an example of a complex interactome, in Figure 2, a DTN generated from DrugBank data sets is shown. The network displays the interactions between 1636 approved small molecule drugs and 1991 human protein targets; the edges represent 7521 unique interactions. Drugs (circles) and targets (diamonds) are connected if an interaction between them is reported in the database. As it is evident from the picture, the network includes a large connected component constituted by 3368 nodes, 1510 of which are drugs. It may be noted that a relevant number of them cluster in a tightly interconnected community comprising mostly neurological (light pink), cardiovascular (brown), and respiratory system (aquamarine) drugs. The analysis of this kind of network provides a global picture of the molecular pharmacological space and might help to identify trends or possible areas of future development in drug research. An example thereof can be found in the paper by Yildirim et al.^64^ Clearly, these models cannot be definitive, and they evolve as new knowledge adds to the database. Starting from this consideration, in the next section, a further step into the use of networks in drug discovery will be illustrated.

## 4 Network-Based Inferences

Going back to the idea that networks are simplified representations of complex systems, it is plausible to recognize that they are not definitive models, but they can evolve as new information becomes available. In other words, when dealing with networks, one has to do with the problem of missing information, a common situation in the study of biological systems, where the difficulty of obtaining experimental evidence of interactions makes the network inherently incomplete but where, on the other hand, new knowledge is continuously added. This issue is quite basic in network theory and pertains to the network reconstruction scope^69^ in the general context of the network statistical physics.^11,70^ In a tighter perspective, however, the possibility of inferring missing links in a network, which is the prediction of a new link between two yet unconnected nodes, is of more practical and immediate interest. Actually, a number of methods of link prediction are available that find wide application, e.g., in social or information networks, where predicting the possible association between individuals or documents, respectively, can be very useful.^71^

### 4.1 Link Prediction Methods

Focusing on drugs, one of the hottest issues in medicine today is the identification of disease-related genes, so much so that considerable experimental efforts are carried out to pursue this goal. Applying network modeling in this field has produced a relevant amount of knowledge so far, and many examples thereof regard the network-based identification of targets (see, for example, ref (72) in the framework of precision medicine) for the elucidation of mechanisms of action, for drug repurposing, or for the discovery of new drugs. In practice, one attempts to predict potential drug–target interactions (DTIs) using one of the numerous available prediction methods, often borrowed from such distant fields as social sciences, communication networking, economy and finance, and so on. The goal is to produce a list of potential DTIs and rank them based on some predefined metrics. The starting point is the construction of a *heterogeneous* network on which to run a link prediction algorithm. Generally, in these cases, the heterogeneous network integrates available information on drugs, targets, and drug–target interactions obtained from different databases. Given the availability of the data, the key steps in these methods are (1) the calculation of the drug–drug and target–target similarities and (2) the application of a drug–target association inference method. As regards the former, after the initial simple use of fingerprints and primary sequences to compare drug molecules and proteins, respectively, more complex and information rich similarity metrics^73^ have been devised in order to take into account also the information coming from known drug–target interactions,^74^ as well as protein and network topological information,^75^ eventually elaborated through ML.^76^ On the methods side, network-based prediction approaches for DTIs vary broadly, often depending on the user’s preference or expertise, but the most popular ones are derived from either recommendation^77^ or network propagation^78^ algorithms, both belonging to the class of the so-called similarity-based algorithms.^71^ The methods based on recommendation algorithms aim at predicting a node’s preferences for unconnected nodes based on previously calculated similarity scores (a technique also called collaborative filtering). This approach was applied by Cheng et al.,^74^ who developed the network-based inference (NBI) method for the prediction of novel DTIs based on a bipartite drug–target graph built from an adjacency matrix obtained from known drug–target interactions. Alaimo et al.^79^ enhanced this method by integrating into the model further domain-dependent biological knowledge, which is drug–drug and target–target similarity measures. On the other hand, under the framework of network propagation algorithms several methods are included that work by simulating the spread of information across the network starting from seed nodes. The most famous one is the Google page rank algorithm^80^ that uses the random walk through Web pages to calculate their importance. In DTI prediction, the random walk with restart (RWR) variant has been developed and successfully applied to drug–target heterogeneous networks.^81,82^ The output of these calculations is a ranked list of probabilities of drug–target association. Further methods based on network propagation have recently been proposed, among which those developed by Sharan and co-workers deserve mention, as they are particularly versatile and applicable also in the personalized medicine setting.^83,84^

### 4.2 Applications to Drug Repurposing

**Figure 3:**
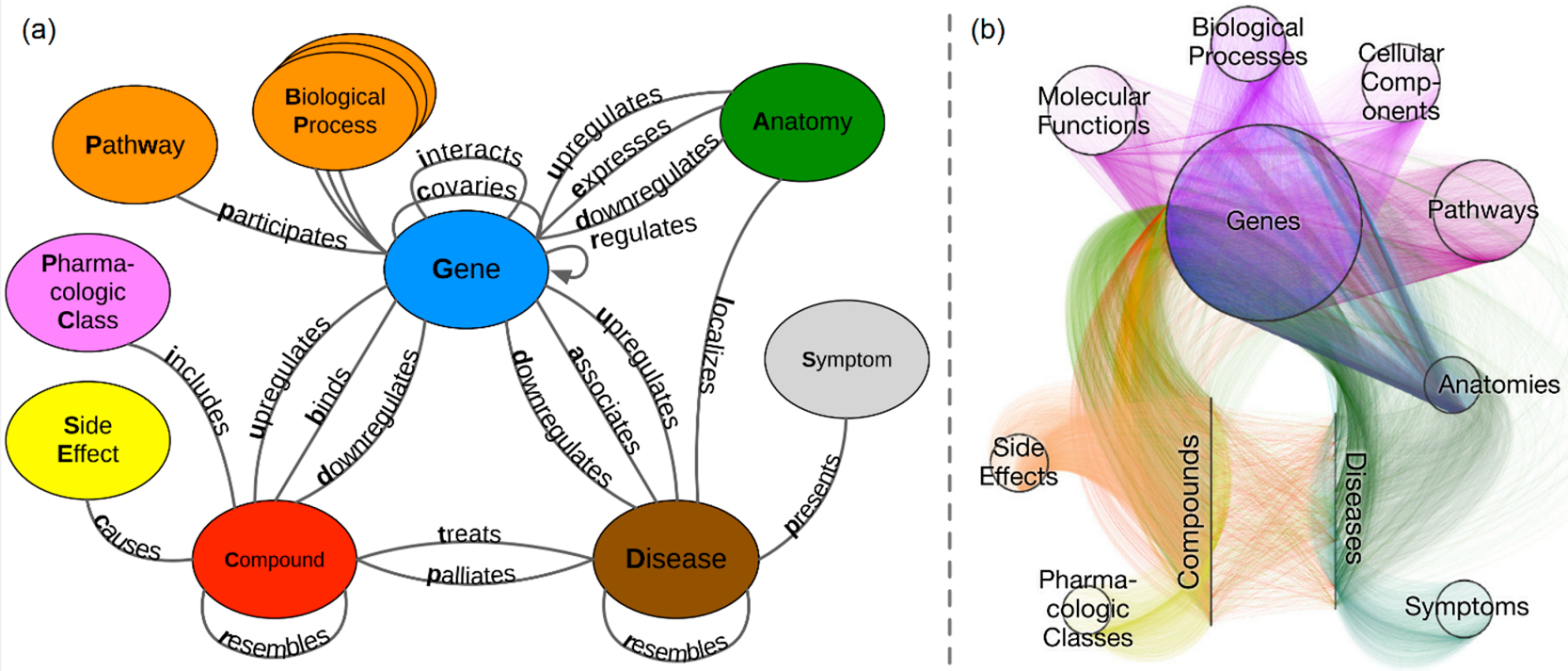
Hetionet version 1.0. (a) Metagraph showing the types of nodes used to build the network and the types of links defined to connect the nodes. A detailed description of the meaning of each link type as well as the sources of information used to collect the nodes and to draw the edges is reported in ref (85). (b) Visualization of the whole heterogeneous network. Nodes of the same type are grouped within circles, and links are colored by type. This Figure 3 is reproduced from Figure 1 of HimmelsteinD. S.; LizeeA.; HesslerC.; BrueggemanL.; ChenS. L.; HadleyD.; GreenA.; KhankhanianP.; BaranziniS. E.; 2017; eLife10.7554/eLife.26726PMC564042528936969 (ref (85)), published under the Creative Commons Attribution 4.0 International Public License (CC BY 4.0; https://creativecommons.org/licenses/by/4.0/).

As an illustrative example of the impact a network-based approach can have on drug repurposing, here we briefly describe a study called Project Repethio (https://think-lab.github.io/p/rephetio/) recently published as a research article on *eLife*.^85^ In this work, the authors report the construction of a heterogeneous network to capture the connections among drugs and diseases (Hetionet version 1.0, https://neo4j.het.io/browser/) and its use to predict new drug/disease associations as probabilities of treatment for the repositioning of known drugs. Hetionet integrates data from public sources and is composed of ∼50 000 nodes of 11 types linked by ∼2.25 million of edges of 24 types. In Figure 3a, the conceptual scheme of the network is shown (a metagraph, i.e., a graph in which the nodes are sets of objects, and the edges connect the sets^86^), indicating types of nodes and links, and in Figure 3b, the whole network is visualized with the nodes grouped by type within circles, and the links are color-coded by type.

In order to extract disease treatment predictions from the drug–disease connections in the network, the authors implemented a ML procedure (logistic regression) trained on a previously compiled gold standard of 755 disease-modifying indications. These known treatments were the positive data, while ∼30 000 nontreatments were used as negatives. In short, the algorithm learns which types of compound-disease paths (metapaths) discriminate between treatments and nontreatments, and on this basis, it predicts new drug–disease associations. For 1538 compounds and 136 diseases, 209 168 associations were prioritized, and the authors provided illustrative validation through literature data and output analysis for two of them regarding nicotine dependence and epilepsy. In our opinion, the Repethio project gives a clear idea of how network-based data analysis can impact drug research, also considering the suitability of this kind of approaches to be interfaced with the powerful ML methods for features selection and prediction. Moreover, it is an example of the use of publicly available data integrated into an online platform that in turn is open to users who can access it and take advantage for their local purposes of a time- and resource-intensive assembly and integration work. We envisage that the more research data become available for the public domain, the more frequent initiatives of this kind develop, leveraging in full the combined potential of big data and network science.

Noteworthy, the variety of network-based DTI prediction methods is growing constantly in these years, and the integration with ML-based tools is frequent, as shown also by the case illustrated above. Interested readers can refer to the numerous recent reviews on the argument, e.g., like ref (87) (see also Discussion). Another direction of development is the consideration of a further source of information in the construction of the heterogeneous network, namely, diseases. This results in the building of tripartite networks, wherein drug–disease and target (gene)–disease associations are included, thus adding a further layer to the knowledge base on which predictions are calculated.^88−90^ For instance, Zong et al.^89^ proposed a DTIs prediction method based on the use of a tripartite network called linked tripartite network (LTN), where drugs, targets, and diseases are nodes connected by drug–target, drug–disease, and target–disease associations. In practice, they merged a bipartite network built from the drug–target associations from DrugBank (Table 1) with the drug–disease and gene–disease associations from the diseasome published by Goh et al.^60^ to obtain the LTN containing 1452 diseases, 8201 drug–disease associations, and 1684 target–disease associations. The method showed high efficiency in the prediction of the associations between drugs and targets within the network, but it was not able to extend the predictivity to new drugs or targets not included in the model.

## 5 Network Dynamics

A step forward in the application of network science in drug research can be taken if we consider the possibility of modeling the time evolution of networks, that is, network dynamics. To appreciate the prospective importance of this field for future drug discovery, it is necessary to briefly introduce Boolean networks that were proposed in 1969 by S. Kauffman in the context of a general hypothesis aimed at accounting for the regulatory circuits controlling homeostasis and differentiation in cells.^91,92^ Interested readers might refer to the thoughtful review by B. Drossel.^93^

### 5.1 Boolean Networks

**Figure 4:**
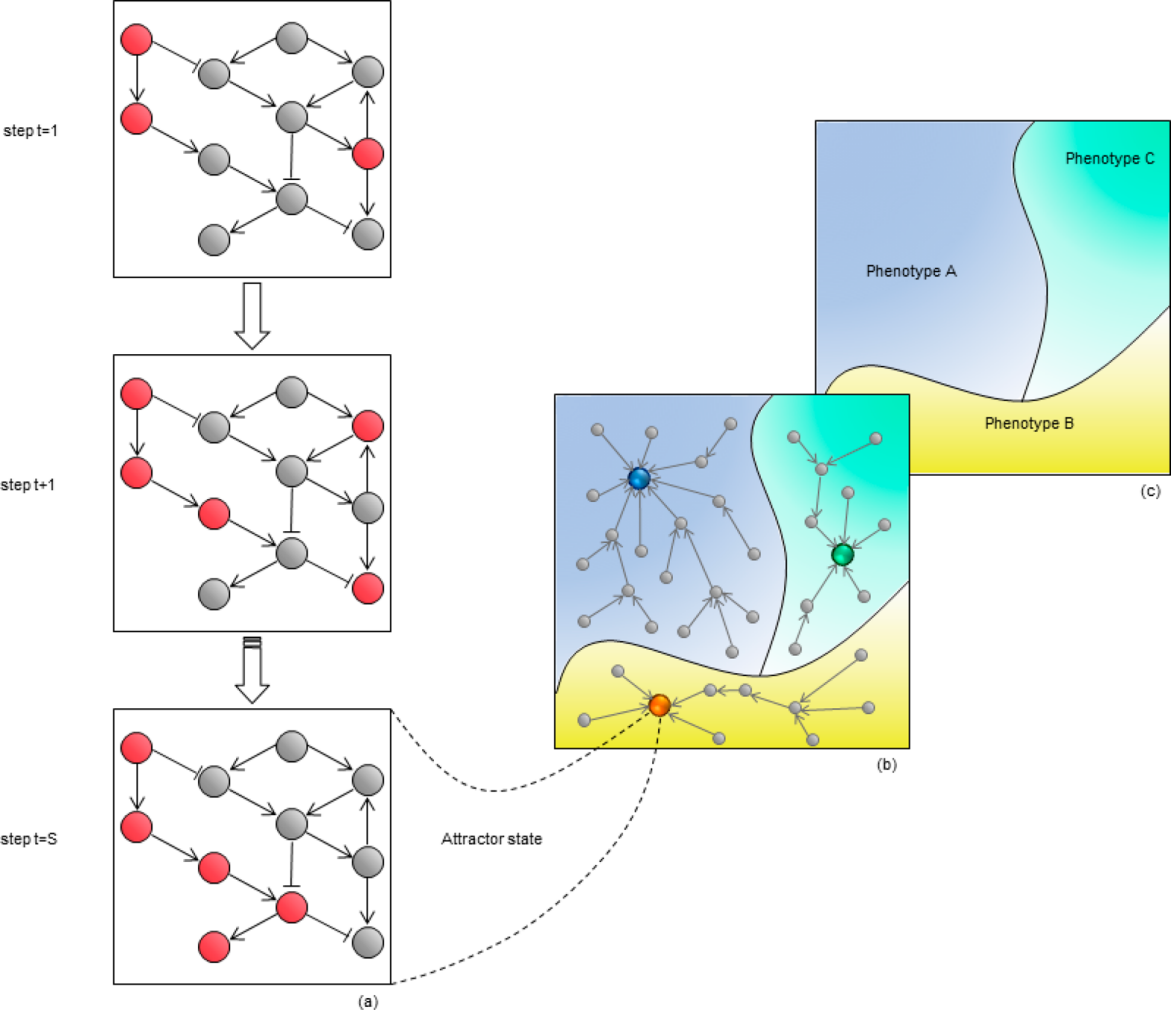
Boolean network dynamics. (a) Nodes are colored red or gray based on their “on” or “off” state, respectively. The stepwise evolution of the interactions between nodes determines some sequential steps that are calculated based on a set of rules. The stable state of the network at time step *t* = S represents an attractor. (b) Attractor landscape. Gray circles represent network states, colored circles represent attractor states. The landscape contains all the possible network states. The sets of states that converge toward an attractor form the basin of that attractor (colored areas). (c) The basins of attractors can be associated with cell phenotypes, and the gene states of the attractors determine the nature of the phenotype.

Boolean networks are directed networks, built in such a way where nodes are genes and links represent the functional connections between them; each gene can be “on” or “off”, and a set of rules or update functions are associated with each node to define the state of the gene at subsequent time steps. The dynamics of the system is calculated starting from input gene(s) by updating simultaneously at each discrete time step all gene states based on the predefined rules. Given the way in which it was built, a Boolean system is deterministic and has a finite number of initial network states (2^*N*^, where *N* is the number of genes, and 2 refers to the two states on and off). After a number of iterations (time steps), it will reach a stable situation that can be a fixed point or a self-looping circle: such a network state can be accessed following different network state sequences (trajectories), and it seems to attract the system to it, being therefore defined an *attractor* (Figure 4a). Several attractors may exist for a single Boolean network, and the ensemble of them together with the trajectories from the initial to the attractor states gives rise to the *attractor landscape* (Figure 4b). It may be noted that a Boolean network can simulate the dynamical state of a system in normal conditions, but it might also be perturbed by modifying the update rules, i.e., setting on or off some nodes. It has been shown that the attractor states correspond to the cellular phenotypes in response to external stimuli (see below), and consequently, exploring the attractor landscape leads to the definition of the *phenotype landscape* (Figure 4c), where a phenotype includes all the network states that lead the system to the same attractor(s).

As a final observation on this kind of models, we point out a claim made by Kauffman in a recent paper, where he revisited his initial hypothesis: answering the question “What is a cell type?”, he wrote “We conclude that there is good evidence that cell types are high dimensional attractors in the dynamics of the genetic regulatory network.”^94^ This means that Boolean networks can be considered as simple cell models able to simulate the dynamic cell behavior in response to both physiological and external stimuli and to predict the cell fate in terms of phenotypic output. In other words, we might predict cellular responses to gene activation/inactivation (such as proliferation, apoptosis, cell cycle arrest, and so on) by calculating the attractors characterizing the attractor landscape (Figure 4b and Figure 4c). By the way, Huang et al. in a beautiful paper were the first to experimentally demonstrate that an attractor state corresponds to a unique cell phenotype that can be reached via different trajectories (gene expression patterns) in response to different perturbations.^95^

### 5.2 Network Dynamics and Drug Discovery

The possible contributions of this network dynamics modeling approach to the field of drug discovery are manifold, as illustrated in the review by Bloomingdale et al.^96^ First of all, simulating transcriptional regulation networks can be useful in view of the possibility of postulating new drug targets. For example, De Anda-Jauregui et al.^97^ built a simplified Boolean model of the estrogen receptor regulatory network and ran dynamics simulations in both unperturbed and perturbed mode. To simulate the perturbations of the network, the authors systematically overexpressed (on) and knocked out (off) all genes, both singularly and in combination. The results of the attractor landscape analysis pointed out some known gene expression regulators as the proteins chiefly involved in the altered proliferative state, thus demonstrating the suitability of the approach for the exploration of cancer-related systems in the search for new drug targets.

Incidentally, it must be noted that to obtain the attractor landscape, the state space of the system should in principle be fully explored by carrying out dynamics simulations starting from all the possible initial states (2^*N*^; see above). This is a computationally expensive task, and some methods have been developed to reduce the size of the network while preserving the properties of the system.^96^

As a further example of the applications of network dynamics, we cite here the work by Choi et al.,^98^ who studied the attractor landscape of the p53 regulatory network and used their analysis to identify druggable targets and putative drug combinations. The simulations allowed them to identify critical nodes in the p53 network able to drive normal and breast cancer MCF7 cells toward proliferation, cell cycle arrest, or cell death (the attractors). Then, on the basis of this information, the authors tested different putative therapeutic interventions (simulated as node or link deletions) aimed at pushing MCF7 cells toward apoptosis. They found and experimentally confirmed that the combined treatment with nutlin-3 (Mdm2-p53 PPI disruptor) and Wip1 inhibitor resulted in enhanced cell death, even in the absence of DNA damage. In a subsequent paper, the same group extended their approach to a panel of 83 human cancer cell lines, for which they mapped the genomic alterations of the p53 network. This allowed them to define 45 differently wired p53 networks that were then submitted to the attractor landscape analysis to identify cancer-specific therapeutic interventions.^99^

Despite the above-mentioned cases, network dynamics is still an underexplored field in the context of drug research, but its promises make it worthy of great attention, particularly if we consider its suitability to the simulation of complex GRNs, e.g., like those involved in carcinogenesis.^100^ In this regard, it is worth mentioning Enrico Capobianco’s observation that “cell state dynamics could be studied by discrete-time Markov models”,^100^ a statement that foreshadows a bridge toward some advanced computational approaches in drug design. Actually, in the molecular simulations setting, Markov state models (MSMs) are currently used to investigate the kinetic aspects of protein molecular dynamics,^101,102^ and their applications in GRN dynamics studies are also known.^103^ Indeed, the possibility to use a common mathematical formalism to describe the dynamics of such different systems as a protein and a GRN opens a formidable perspective in view of the multiscale modeling of biological systems for drug discovery.

## 6 Discussion

Networks and network analysis tools are rather widespread in biology/chemical biology, not so in chemistry/medicinal chemistry. In any case, if we consider the broad field of drug research, we see an increased use of this kind of *in silico* modeling approach. In our view, medicinal chemists should be aware of this and take every opportunity to enter this fascinating field, the same way as when the LFER/QSAR paradigm^104^ was introduced by Corwin Hansch.

In the design of a new drug, the scenario depicted by a system-based network model can be very useful and illuminating, for both practical and theoretical reasons. As regards the former, in perspective, the process of identification and selection of new drug candidates based on a network representation of the target biosystem (“network-driven drug discovery”^105^) might be viewed as a kind of “*in silico* integrated screening”, in some way simulating and expanding the experimental phenotypic screening (e.g., see Turbine, an artificial intelligence platform for studying cancer, https://turbine.ai). In fact, on the basis of the available knowledge, one can build the network of interactions governing the cell behavior (an *in silico* cell) and identify those interventions able to drive the cell toward the desired fate. The network might include several layers of description, from the molecular to the *in vivo* ones and even above. Considering the already mentioned growing production of molecular, -omic, clinical, etc. data, it seems reasonable to foresee the possibility of realizing the “vertical model integration” proposed by Xie et al.^7^ through the construction of multipartite networks. This “*in silico* pharmacology/systems biology continuum”^106^ might allow researchers to take into consideration simultaneously most of the effects determining a drug’s action process, thus increasing the reliability of predictions and lowering the costs of drug candidates selection.

On another side, from a theoretical point of view, taking a network approach to investigate human diseases means considering the intrinsic complexity of living organisms, which is nowadays accepted as a mandatory standpoint to confront pathologies.^68^ Coming to drugs and limiting ourselves to the pharmacological way of tackling the disease problem, the holistic view of the target system might help to devise new strategies of design. Peter Csermely gave an interesting example of this by proposing two strategies of network-based drug identification: the central hit strategy (CHS) and the network influence strategy (NIS).^14^ In the first one (not new, actually), the aim is to damage a cellular network by hitting a critical node (as in antiinfectious or anticancer therapies), while in the second one, drug(s) should hit influential nodes in order to rewire the diseased network toward its normal state. Of course, key points in the application of these strategies are the analysis of the network topology, and, in the case of NIS, also the network dynamics. Moreover, Csermely convincingly described how such a way of tackling drug-related issues, while taking into account the complexity of the system, might well incorporate current operative concepts, e.g., like PPI inhibitors, multitarget drugs, allosteric drugs, and hit/lead development. In this sense, a network-based view allows one to expand both the starting point and the landscape from which the drug discovery process is considered. Not being merely a technological improvement, it could eventually lead to devising alternative paradigms of pharmacological intervention.

A further theme worthy of discussion is how the network modeling approach can be integrated with the well-developed computational techniques currently employed in drug design/discovery, namely, molecular modeling and simulations, and ML. Indeed, ML and deep learning methods are already widely used in computational drug discovery,^107,108^ and they are perfectly suited to integrate in both the network construction techniques and the network-based prediction approaches. Examples thereof can be found, for example, in the works of Yamanishi et al.^109^ (application of a kernel regression method to build a pharmacological space by integrating a chemical space and a genomic space) or Mei et al.^110^ (use of a supervised bipartite model incorporating additional training from neighbors in order to predict DTIs for drugs and targets not included in the network). In a recent paper, Zhou et al.^111^ proposed a classification for the computational models used for DTI prediction comprising both network-based methods (i.e., the similarity-based algorithms outlined in section 4.1) that employ algorithms derived from network theory, and ML-based methods that belong to the realm of statistical learning.^112^ Taking this point of view, we can depict a drug discovery scenario where, given a complex system to be interpreted or on which predictions have to be made, one can rely on a rich toolbox of methods that are purely network-based (section 4.1) or ML-based^112^ or a merge of both.^87^

Regarding the inclusion in networks of the atomic-level 3D information obtained by molecular modeling and simulations, this issue has to be carefully considered in order to appreciate its potential in drug discovery. In fact, if we had to do with a drug–target network or a PPI network, why not consider the protein nodes as conformational ensembles? In this way, it might become straightforward to expand each node into the set of protein conformations. In such a case, this would lead to the obtainment of a 3D interactome that could take into account the possibility of a protein binding different partners with different conformations. In an analogous way, in a bipartite drug–target network, different conformations of the same protein could bind different drugs, and should the network describe this feature, it would increase substantially the informative content of the model and its ability to predict potential DTIs. If we imagine such a “3D network model” to be used for the selection of drug candidates, it might constitute a bridge between 3D agnostic network-based prediction and classical target-based molecular design, with a great synergistic potential in terms of efficiency. In a recent commentary,^113^ Mih and Palsson discussed the perspectives of a similar scenario in systems biology. They presented several studies where structural information on ligands and proteins were included in genome-scale metabolic network models, thus allowing a more detailed level of description of such systems.

Finally, limitations and open challenges of the network-based approach to drug discovery have to be highlighted. The first problem has already been pointed out in the section on data and databases and pertains to the quality of the data of whatever type they are and whatever source they come from. Moreover, biological data may be incomplete, biased, or sparse, and the languages used to build the databases may be different and/or incompatible. All this limits severely the possibility of even building a network. The second challenge has to do with the dimensions of the data and consequently of the network. Depending on number of nodes and node degrees, the number of links to calculate can increase enormously, and this again limits the possibility of building or analyzing the network, even though some tools allow one to deal with up to millions of nodes and edges (see the review by Pavlopoulos et al.^42^). However, as the analysis becomes more detailed or complex (e.g., network dynamics), the computational demand becomes prohibitive. A way to overcome the computational problems is to distribute the workload on the cloud, a choice that seems the best technological option presently available, while waiting for the effective accessibility of quantum computers.

## 7 Conclusion

In his “General Systems Theory” book, in 1969, Ludwig von Bertalanffy wrote “In one way or another, we are forced to deal with complexities, with ‘wholes’ or ‘systems’, in all fields of knowledge. This implies a basic re-orientation in scientific thinking.”^114^ This statement fits perfectly the idea at the basis of this paper about the way drug research should be approached. Nowadays, we are well aware that the research paradigm in the pharmaceutical field has changed in this sense, and what is presented here is an *in silico* framework coherent with the new way of thinking drug discovery. By no means does this imply that the classical computational approaches to drug design should be replaced by network theory but on the contrary that they might be more efficiently employed if integrated in a network context. With a careful awareness to the open challenges outlined above, network-based discovery methods will be key players in the next decades drug research.

## Supporting Information Available

Short presentation of the basic concepts of network theory (PDF)

## Abbreviations Used

QSAR: quantitative structure–activity relationship
ML: machine learning
NAR: *Nucleic Acid Research* (journal)
EHR: electronic health record
CSN: chemical space network
SAR: structure–activity relationship
PSN: protein structure network
GRN: gene regulatory network
PPI: protein–protein interaction
DTI: drug–target interaction
NBI: network-based inference
RWR: random walk with restart
MSM: Markov state model
LFER: linear free-energy relationship
CHS: central hit strategy
NIS: network influence strategy
