# Supplementary material for: Drug Research Meets Network Science: Where Are We?
Source: J Med Chem. 2020 Apr 27;63(16):8653–66. doi: 10.1021/acs.jmedchem.9b01989 (PMC8007104; doi:10.1021/acs.jmedchem.9b01989)
Supplement: Supplementary file 1 — jm9b01989_si_001.pdf [file jm9b01989_si_001.pdf]

Drug research meets network science: where are we?

Maurizio Recanatini and Chiara Cabrelle*

Department of Pharmacy and Biotechnology, Alma Mater Studiorum – University of Bologna, Via Belmeloro 6, I-40126 Bologna, Italy

Supporting Information

Basics on networks and network analysis

1. Basic concepts of graph theory

The analysis of networks requires mathematical tools derived from the graph theory. By definition, a graph G consists of a set of *nodes* or *vertices* (V) and a set of *links* or *edges* (E) between vertices, formally expressed by G = (V, E). Each edge $e \in$ E joins two vertices i and $j \in$ V that are called its endpoints; this edge is written as $e = \langle i, j \rangle$, and vertices i and j joined by edge e are called adjacent. According to this description, two vertices i and j could be joined by multiple edges forming a set of edges having i and j as endpoints. A graph without multiple edges or loops, *e. g.*, edges joining the same vertices, is called simple graph. A simple graph in which each vertex is adjacent to all the other vertices is named a complete graph. In graph terminology, all vertices adjacent to a vertex i define the neighbour set N(i). The *degree* of a vertex i, indicated as $k(i)$, is the number of edges joining it to the other vertices.

2. Graph types

A graph of the type G = (V, E), in which an edge $e = \langle i, j \rangle$ is defined by an unordered pair of vertices, is called an *undirected graph*. This does not always provide a satisfying representation of the network under study, and accordingly, other graph types can be used.

A *directed graph* or digraph D = (V, E) is defined by a set of vertices V and a set of edges E of ordered pairs of vertices such that each edge *e* has a direction. Directed graphs are used in biology to represent systems involving sequential interactions between the elements, like the well known gene regulatory networks.

A *weighted graph* consists of a graph in which each edge has an associated real number called its weight w_{ij}. The w_{ij} of the edge *e* connecting vertices *i* and *j* represents the intensity of the interaction. An application of this graph type is represented by the PARP inhibitors network reported in Figure 1 of the main article, where the edge thickness is associated with the molecule similarity values.

Another important class of graphs is that of *multi-partite graphs*, among which the bipartite graphs are the most widely used in drug discovery applications. A *bipartite graph* is a graph G = (V, E) in which the set of vertices V is partitioned into two subsets V_1 and V_2, such that each edge connects only vertices of different type. To compress the information of a bipartite network, the corresponding two mono-partite networks can be projected. In the projections, two nodes are linked if they share at least one common neighbor in the bipartite network. As an example of a bipartite graph, in Figure 2 of the main article, the drug target network from DrugBank is shown with drugs and targets representing the two distinct sets of nodes.

A schematic representation of the mentioned graph types is shown in Figure S1.

3. Data structure

It is essential to consider how to store the data underlying graph representations, that may appear tricky especially for large graphs. Taking into consideration computer readable format, the most common data structure is the *adjacency matrix*.

The adjacency matrix A for an undirected graph G = (V, E) is a symmetric |V|x|V| matrix with A[*i*,*j*] = A[*j*,*i*] for all *i* and *j* vertices. For a simple graph, if an edge joins *i* and *j* vertices, the entry A[*i*,*j*] = 1, otherwise A[*i*,*j*] = 0 and A[*i*,*i*] = 0. For a directed graph the adjacency matrix is not symmetric so A[*i*,*j*] ≠ A[*j*,*i*], while in the case of weighted graphs, for an edge *e* with weight w_{ij} between *i* and *j*

vertices, the entries of the adjacency matrix are equal to w_{ij} so A[*i*,*j*] = w_{ij}. For bipartite graphs G = (V_1, V_2, E) with two sets of vertices V_1 and V_2 the biadjacency matrix is represented by a $|V_1|$x$|V_2|$ matrix in which B[*i*,*j*] equals 1 when an edge *e* between *i* and *j* vertices belonging to two diverse sets is present, and 0 otherwise. In Figure S1, a schematic illustration of simple graphs and their corresponding adjacency matrices is reported.

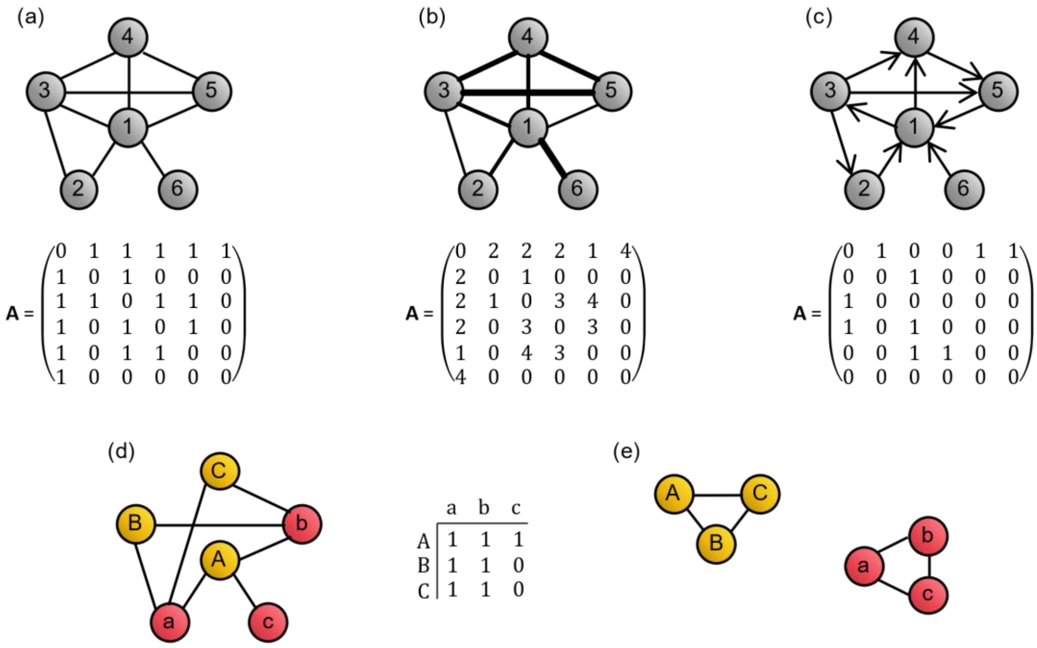

Figure S1. Network types and relative adjacency matrices. The figure shows (a) a simple undirected graph with its symmetric binary adjacency matrix; (b) a weighted graph in which the thickness of the edges represent weights reported in the adjacency matrix; (c) a directed graph with non-symmetric edges and its adjacency matrix; (d) bipartite graph in which nodes belonging to two diverse sets are displayed with different colors and (e) the corresponding two mono-partite graphs.

4. Network analysis

Network theory has developed some basic parameters that refer either to the whole network or to single nodes, and that enable to characterize the network and to analyze its features.

Taking into consideration node properties, the fundamental one is the node *degree* together with the *degree distribution*.

As regards the global network, besides the total numbers of nodes and edges, it is often important to identify *communities* in turn giving rise to *clusters* or *cliques*. Such communities characterize the global structure of the network and may lead to the identification of modules often endowed of functional meaning.

4.1. Degree and degree distribution

As mentioned before, the vertex *degree* indicates the number of edges of a vertex i denoted as *k(i).* For undirected graphs G = (V, E) with N vertices, the degree *k(i)* for vertex *i* is the sum of the values in its row or column *i* of the adjacency matrix A: $k(i) = \sum_{j=1}^{N} \mathrm{A}[i,j]$. In a directed graph, given A[*i*,*j*] = 1 for the edge pointing from vertex *j* to vertex *i*, for the vertex *i* the in-degree can be defined as the number of edges incoming to i and the out-degree, as the number of edges outcoming from i. The out-degree of vertex *i* is the sum of the column *i* values: $k_{out}(i) = \sum_{j=1}^{N} \mathrm{A}[j,i]$, whereas the in-degree is the sum of the row *i* values: $k_{in}(i) = \sum_{j=1}^{N} \mathrm{A}[i,j]$.

Considering the vertex degree provides a way to identify *hubs*, *i.e.*, high degree vertices that assume a pivotal role within the graph, and the removal of which disconnects the network.

The vertex *degree distribution p*(*k*), that is, the fraction of vertices with degree *k*, accounts for the probability that a randomly chosen vertex has degree *k*.

The graphical representation of degree and degree distribution for a simple graph is shown in Figure S2.

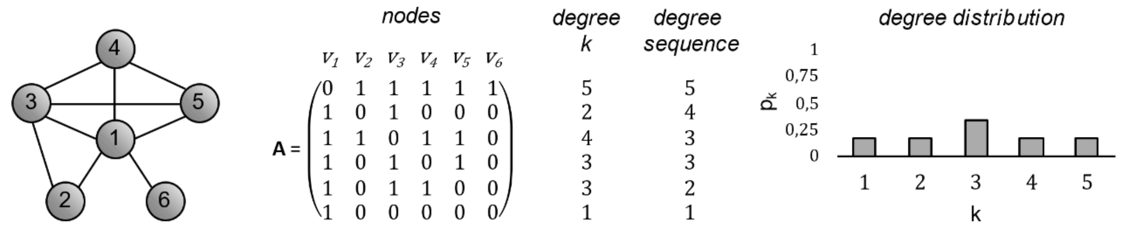

Figure S2. Degree and degree distribution. The figure illustrates for a simple undirected graph with its adjacency matrix how degree k of each vertex can be easily calculated by row or column sum; the degree sequence (a vector of all vertices degrees k), and the degree distribution are also shown.

4.2. Connectivity

The potential of network analysis lies also in the opportunity to study how one node can be reached from another one through a route that highlights the relationship between them. Herein, we briefly recall the terminology related to connectivity.

A *walk* is a sequence of vertices and edges between two vertices from i to j. A *cycle* is a closed walk with all distinct vertices except for $i = j$ and all distinct vertices. A *path* is a walk in which all vertices and all edges are distinct and its length corresponds to the number of edges forming the path. The path between vertices i and j with the minimum number of edges is the *shortest path*, and the distance d_{ij} from i to j is the length of the shortest path. It is possible to find more than one shortest path between two vertices. The *diameter* of the graph (d_{max}) is the longest shortest path in the graph and *the average path length* ($\langle d \rangle$) is the average of the shortest path lengths between all pairs of vertices.

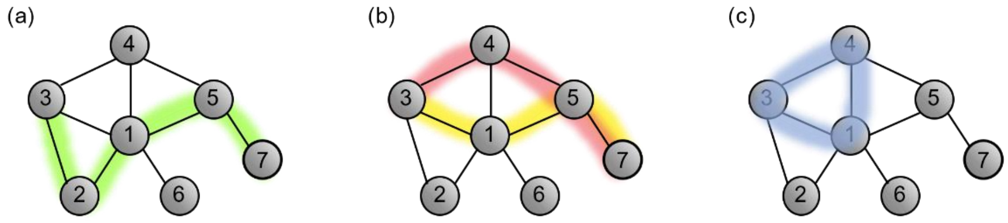

Figure S3. Connectivity. The figure exemplifies for a simple graph (a) a path of length four between vertices 3 and 7, (b) the two shortest paths between the same vertices with distance $d_{37} = 3$, and (c) a cycle of length three.

This terminology is useful to describe the *connectivity* in an undirected network such that if a path between two vertices i and j exists, they are said to be connected, otherwise their distance d_{ij} equals ∞ and they are referred to as disconnected. In the same way, a network is connected if all vertices

pairs are connected, as opposed to a disconnected network that is composed by more than one component such that almost one vertices pair is at infinite distance.

4.3. Subgraphs and cliques

Sometimes one needs to define a graph within the graph and hence a subgroup of vertices and edges. Basically, a *subgraph* of graph G = (V, E) is a graph H that contains a subset of vertices V(H) belonging to V(G), and a subset of edges E(H) belonging to E(G). It is worth mentioning also the induced subgraph that contains all possible edges linking the vertices V(H) in the original graph G. A complete subgraph is termed a *clique*. The maximum clique is the largest fully connected subgraph of G not to be confused with the maximal clique, a clique that cannot be expanded and hence it is not contained in a larger clique.

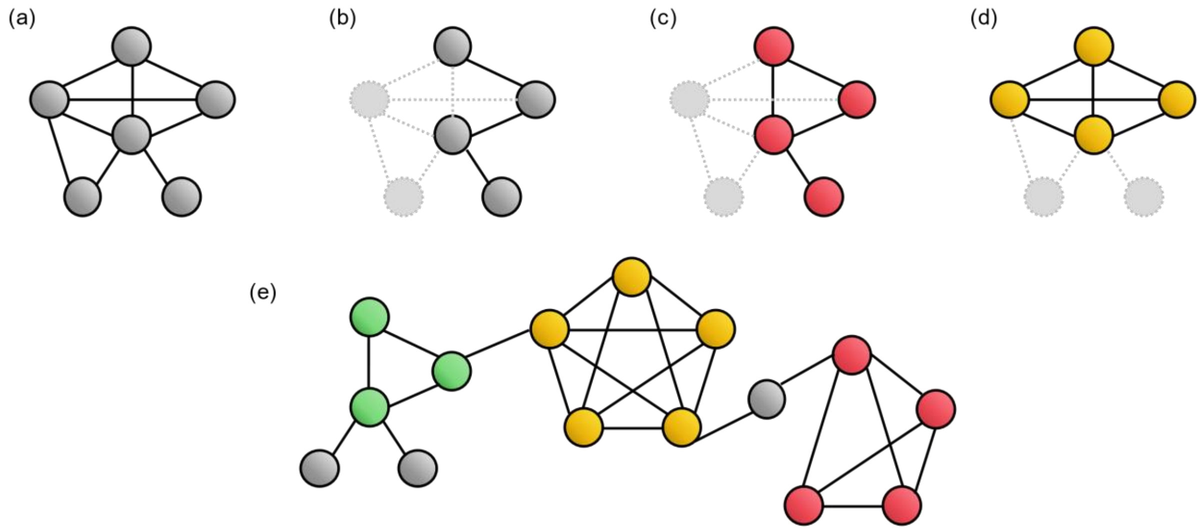

Figure S4. Subgraphs and cliques. The figure shows (a) a simple graph and (b) an example of subgraph with four vertices (dark grey) and three edges (black); (c) an induced subgraph that differs from (b) because it includes all the edges of the original graph linking the subset of vertices of the subgraph (red); (d) a clique (green); (e) a connected graph with three cliques: the green one is a 3-clique, *i.e.* a cycle, the red one is a 4-clique and the yellow one is a 5-clique, the latter being the maximum clique and the other two being maximal cliques.

4.4. Clustering coefficient

The importance of a node in the network is estimated by several parameters related to its position in the community and the relationships with neighbors: one of such parameters is the *clustering coefficient*. Strictly speaking, the local clustering coefficient describes the microenvironment of a node in term of tendency to make a cluster. By definition, this parameter quantifies the extent to which the neighbours of a vertex *i* are also neighbours to each other. In a simple undirected graph G, the local clustering coefficient of vertex *i* with degree *k(i)* > 1 and number of neighbours n_i is expressed by the formula $C_i = \frac{2e_i}{n_i(n_i-1)}$ where e_i is the number of edges between the n_i neighbours. The clustering coefficient range is $0 < C_i < 1$, so that it is equal to 1 in the case of a clique.

The *average clustering coefficient* of the entire graph G is defined as the average of the clustering coefficients of all the vertices *i* belonging to V(G): $C = \frac{1}{V}\sum_{i \in V} C_i$.

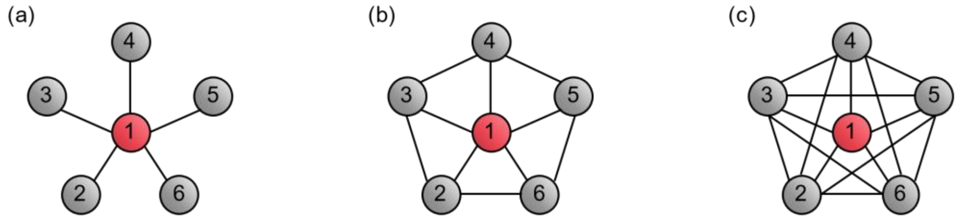

Figure S5. Clustering coefficient. The figure shows graphs with five vertices that differ from each other for the number of edges joining the neighbours of vertex 1 such that its clustering coefficient scales being in (a) $C_1 = 0$, in (b) $C_1 = 0{,}5$ and in (c) $C_1 = 1$ representing a clique.

5. Centrality metrics

Several centrality measures could be taken into consideration, among which we briefly describe the closeness and betweenness centralities.

5.1. Closeness centrality

The *closeness centrality* of a vertex quantifies how close this vertex is to the other vertices in terms of length of the shortest paths, hence of distance. In a connected undirected graph G = (V, E), the

closeness centrality of vertex i is defined by the formula: $c_{cl}(i) = \frac{1}{\sum_{j \in V} d_{ij}}$, where d_{ij} is the distance from vertex i to vertex j. A node with high closeness centrality is a crucial element, since it easily reaches and more directly influences the other nodes of the network.

5.2. Betweenness centrality

The *betweenness centrality* highlights the importance of a vertex as intermediary between the others. The reason why this kind of nodes is important is due to their essentiality for preserving the connectivity: their removal causes the partitioning of the network. So in a connected graph G = (V, E) with i, j and $z \in$ V, σ_{ij} is the number of shortest paths between vertices i and j, and $\sigma_{ij}(z)$ is the number of shortest paths going through z, the betweenness centrality is defined by the formula $c_b(z) = \sum_{i,j \in V(z)} \frac{\sigma ij(z)}{\sigma ij}$, where V($z$) is the set of all pairs i, j such that i, j and z are distinct.

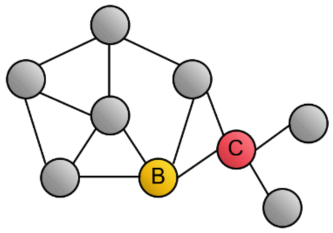

Figure S6. Closeness and betweenness centrality. In the simple graph, vertex B (yellow) has the highest closeness and vertex C (red) has the highest betweenness.

6. Communities

The identification of groups of densely connected vertices within a biological network might be useful to discover functional modules, which can represent sets of proteins or genes involved in a specific cellular process. This idea intuitively captures the concept of a *community*, but a precise mathematical definition lacks. A community might be termed a subgraph whose nodes are tightly connected with each other and poorly connected with the rest of the network. Several methods exist for the identification of communities that can be grouped into two types, the graph partitioning and community detection algorithms. The problem of dividing the vertices of a network into groups is a

well known one in network theory, and the interested readers can refer to specialized texts (see, *e.g.*, M.E.J. Newman, *Networks. An Introduction*, Oxford University Press, Oxford, UK, 2010, Chapt. 11)

7. Network topology and network models

By network topology, we mean the arrangement of the elements in the network that determines its architecture. About global network properties, it is remarkable that the networks we are dealing with in drug research can be generally classified as *scale-free*, and are different from the so-called *random* networks. The most popular random graph model is that due to Erdős and Rényi[1–3] described by G(n, p) with n defining the number of nodes and p being the probability of edges between vertices, so the number m of edges is not fixed. These ensemble of graphs G(n, p) has a binomial degree distribution that for large networks corresponds to a Poisson distribution. Nonetheless, often real networks are characterized by specific structures that do not approximate random graphs, because they imply a topology wherein few nodes are hubs and there is a prevalence of small-degree nodes. The Barabási-Albert model[4] can generate so-called scale-free networks modeling these properties peculiar of many biological networks. In fact, the degree distribution $p(k)$ of a scale-free network does not fit the binomial distribution of random graphs, but can be approximated by a power law distribution $p(k) \sim k^{-\gamma}$, where γ is the degree exponent.

Finally, to examine the topology of a network, one might also consider the property of *assortativity* that takes into account the tendency of a node to link to similar nodes. Therefore, a network can be defined *assortative* if nodes with high degree are joined to nodes which have high degree themselves, and the same for nodes with a low degree that are more likely to connect with other low degree nodes. As opposite, in a *disassortative* network, nodes with high degree are connected to low degree nodes, and vice versa. The assortativity of a network is estimated by considering the degree correlation that can be computed measuring for each node the average neighbor degree.

References

(1) Erdős, P.; Rényi, A. On Random Graphs. *Publ. Math.* **1959**, *6*, 290–297.

(2) Erdős, P.; Rényi, A. On the Evolution of Random Graphs. *Publ. Math. Inst. Hung. Acad. Sci* **1960**, *5*, 17–60.

(3) Erdős, P.; Rényi, A. On the Strength of Connectedness of a Random Graph. *Acta Math. Acad. Sci. Hungaricae* **1964**, *12*, 261–267.

(4) Barabási, A. L.; Albert, R. Emergence of Scaling in Random Networks. *Science* **1999**, *286*, 509–512.
